# Prevalence and genotyping of *Giardia duodenalis* infections in humans in Thailand: a systematic review and meta-analysis

**DOI:** 10.1186/s12879-025-12372-6

**Published:** 2025-12-20

**Authors:** Manas Kotepui, Supaluk Popruk, Kwuntida Uthaisar Kotepui, Frederick Ramirez Masangkay, Kinley Wangdi, Aongart Mahittikorn

**Affiliations:** 1 Medical Technology Program, Faculty of Science, Nakhon Phanom University, Nakhon Phanom, 48000 Thailand; 2 Department of Protozoology, Faculty of Tropical Medicine, Mahidol University, Bangkok, 10400 Thailand; 3 Department of Medical Technology, Faculty of Pharmacy, University of Santo Tomas, Manila, 1008 Philippines; 4 Research Center for the Natural and Applied Sciences, University of Santo Tomas, Manila, 1008 Philippines; 5 HEAL Global Research Centre, Health Research Institute, Faculty of Health, University of Canberra, Bruce, ACT 2617 Australia; 6 National Centre for Epidemiology and Population Health, Australian National University, Canberra, ACT 2601 Australia

**Keywords:** *Giardia*, Giardiasis, Thailand, Systematic review, Meta-analysis

## Abstract

**Background:**

*Giardia duodenalis* remains a neglected parasitic infection in Thailand, with uncertain national prevalence and limited data on genetic assemblages. This systematic review and meta-analysis aimed to estimate the pooled prevalence of *G. duodenalis* infections in Thailand and assess the distribution of assemblages to inform surveillance and control strategies.

**Methods:**

The protocol for this study was registered in PROSPERO (CRD42024629406). A comprehensive search was conducted across six databases: EMBASE, Ovid, PubMed, Scopus, Nursing & Allied Health Premium, and the Thai-Journal Citation Index (TCI). The methodological quality of included studies was assessed using the Joanna Briggs Institute (JBI) critical appraisal checklist for observational studies. A meta-analysis using random-effects models estimated the pooled prevalence of *G. duodenalis* infections and the distribution of assemblages.

**Results:**

Sixty-three studies comprising of 47,989 participants were included. The prevalence of *G. duodenalis* infection varied substantially by diagnostic method, ranging from 1.40% using direct smear microscopy to 1.99% using concentration techniques, 3.66% with culture-based methods, 13.44% with immunoassays, and 8.98% with molecular techniques (*P* < 0.0001). Prevalence also differed geographically, with the highest estimates in Western Thailand (12.22%) and the lowest in Northeastern Thailand (0.54%). Vulnerable groups, including orphans (15.14%) and preschool children (11.73%), carried a disproportionate burden of infection. Among the 240 samples analyzed by molecular methods, Assemblage B was the most common (55.0%), followed by Assemblage A (32.5%), mixed A/B infections (12.08%), and a single case of Assemblage F (0.42%).

**Conclusion:**

*G. duodenalis* remains endemic in Thailand, with transmission concentrated in socially vulnerable groups and regions with higher environmental risk. The predominance of Assemblage B and the underuse of molecular diagnostics highlight critical gaps in understanding transmission pathways. These findings suggest that control strategies in Thailand should prioritize high-risk populations and incorporate molecular epidemiology into routine surveillance to more effectively guide prevention efforts.

**Clinical trial:**

Not applicable.

**Supplementary Information:**

The online version contains supplementary material available at 10.1186/s12879-025-12372-6.

## Introduction

*Giardia duodenalis* (also known as *Giardia lamblia* or *Giardia intestinalis*) is a flagellated protozoan parasite that inhabits the small intestine of humans and various animals, causing giardiasis [1]. Transmission primarily occurs through the fecal-oral route via contaminated water, food, or direct person-to-person contact [1]. *G. duodenalis* infections can be asymptomatic or present with gastrointestinal symptoms such as diarrhea, abdominal cramps, bloating, and malabsorption [2]. These infections are reported globally, with prevalence rates varying by region, population, and diagnostic methods [3]. *G. duodenalis* infections are typically reported in developing countries, where inadequate sanitation, poor hygiene, and contaminated water sources contribute to transmission [2]. *G. duodenalis* is classified into eight [8] genetic assemblages (A–H) based on analyses of the small subunit ribosomal ribonucleic acid (RNA) (SSU rRNA), glutamate dehydrogenase (gdh), triose phosphate isomerase (tpi), and beta-giardin (bg) genes, with assemblages A and B being the primary types that infect humans and wild mammals [4].

A previous meta-analysis revealed that *G. duodenalis* was the most common intestinal parasite in Africa, with estimated prevalence rates exceeding 50% [5]. *G. duodenalis* infections among African children were highest in Niger (65.1%) and were particularly associated with high-risk populations, including iron-deficient children, children with disabilities, human immunodeficiency virus (HIV)-infected children, and displaced children [6]. In Asian populations, the overall prevalence was estimated at 15.1%, with the highest prevalence reported in Tajikistan (26.4%). Within these populations, males showed a higher prevalence rate than females [7].

In Thailand, *G. duodenalis* infections have been documented across different populations, with prevalence varying by region, age group, and detection methods [8–11]. Studies have primarily focused on children [8, 12–14], rural communities [15–17], and immunocompromised individuals [18, 19]. However, comprehensive national data on *G. duodenalis* prevalence, particularly across varying diagnostic methods and population subgroups, remain limited. A systematic review and meta-analysis are essential given the potential health risks associated with *G. duodenalis* infection, particularly among vulnerable populations such as children, immunocompromised individuals, and rural communities. This study aimed to estimate the pooled prevalence of *G. duodenalis* infections in Thailand, analyze trends in prevalence over time, and assess variations across regions, population groups, and diagnostic methods. Additionally, the study aimed to determine the distribution of *G. duodenalis* assemblages in Thailand, thereby enhancing epidemiological understanding and informing targeted public health interventions.

## Methods

### Registration and protocol

This systematic review and meta-analysis were conducted to estimate the prevalence of *G. duodenalis* infections in Thailand. The study followed the Preferred Reporting Items for Systematic Reviews and Meta-Analyses (PRISMA) guidelines [20]. The protocol of this study was registered in PROPERO (CRD42024629406).

### Search strategy

A comprehensive search was conducted across the following databases: EMBASE, Ovid, PubMed, Scopus, Nursing & Allied Health Premium, and the Thai-Journal Citation Index (TCI). The search was designed to include all relevant studies published from inception through December 2024. Keywords included combinations of “*Giardia*”, “Giardiasis”, “*Giardia lamblia*”, “*Giardia intestinalis*”, “*Giardia duodenalis*”, and related terms (Table S1). The reference lists of included studies were also screened to identify additional relevant studies.

### Eligibility criteria

Studies were included if they met the following criteria: (i) reported the occurrence of *G. duodenalis* infections in stool samples from human populations in Thailand; (ii) utilized valid diagnostic methods, such as direct smear, concentration methods, molecular techniques, or immunoassays; and (iii) followed observational study designs, including cross-sectional studies, cohort studies (reporting baseline prevalence), or retrospective descriptive studies. Studies were excluded if they: (i) were conducted outside Thailand; (ii) used non-human samples; (iii) focused solely on diagnostic test performance without reporting prevalence; or (iv) were non-original articles, such as conference abstracts, review articles, or letters to the editor.

### Study selection and data extraction

All identified records were imported into EndNote (version 21.0, Philadelphia, PA), and duplicates were removed. Two independent reviewers (MK, AM) screened the titles and abstracts for relevance. Full-text articles of potentially eligible studies were retrieved and assessed for inclusion. Disagreements were resolved through discussion or consultation with a third reviewer (SP). A standardized data extraction form was used to collect the following information from included studies: study characteristics (e.g., publication year, design, and geographical region), population details (e.g., age group, type of participants), diagnostic methods for *G. duodenalis* detection, and the reported number of *G. duodenalis* infections. Two reviewers (AM and MK) independently performed data extraction, and discrepancies were resolved through consensus.

### Methodological quality assessment

The methodological quality of included studies was assessed using the Joanna Briggs Institute (JBI) critical appraisal checklist for observational studies [21]. Criteria included clear definitions of study settings, valid and reliable measurements, identification of confounding factors, and appropriate statistical analysis.

### Statistical analysis

A meta-analysis to estimate the pooled prevalence of *G. duodenalis* infections was conducted using random-effects models [22]. Prevalence estimates were expressed as percentages with 95% confidence intervals (CIs). Heterogeneity across studies was assessed using the *I²* statistic [23], and potential sources of heterogeneity were explored through subgroup and meta-regression analyses. Subgroups analysis included study periods, geographical regions, study design, participant types, and diagnostic methods. Publication bias was assessed using funnel plots and Egger’s test [24]. All statistical analyses were performed using the meta package in R (version 4.4.2) [25], with RStudio (version 2024.04.2 + 764) [26] serving as the user interface. A *P* value of less than 0.05 was considered statistically significant.

## Results

### Search results

**Fig. 1:**
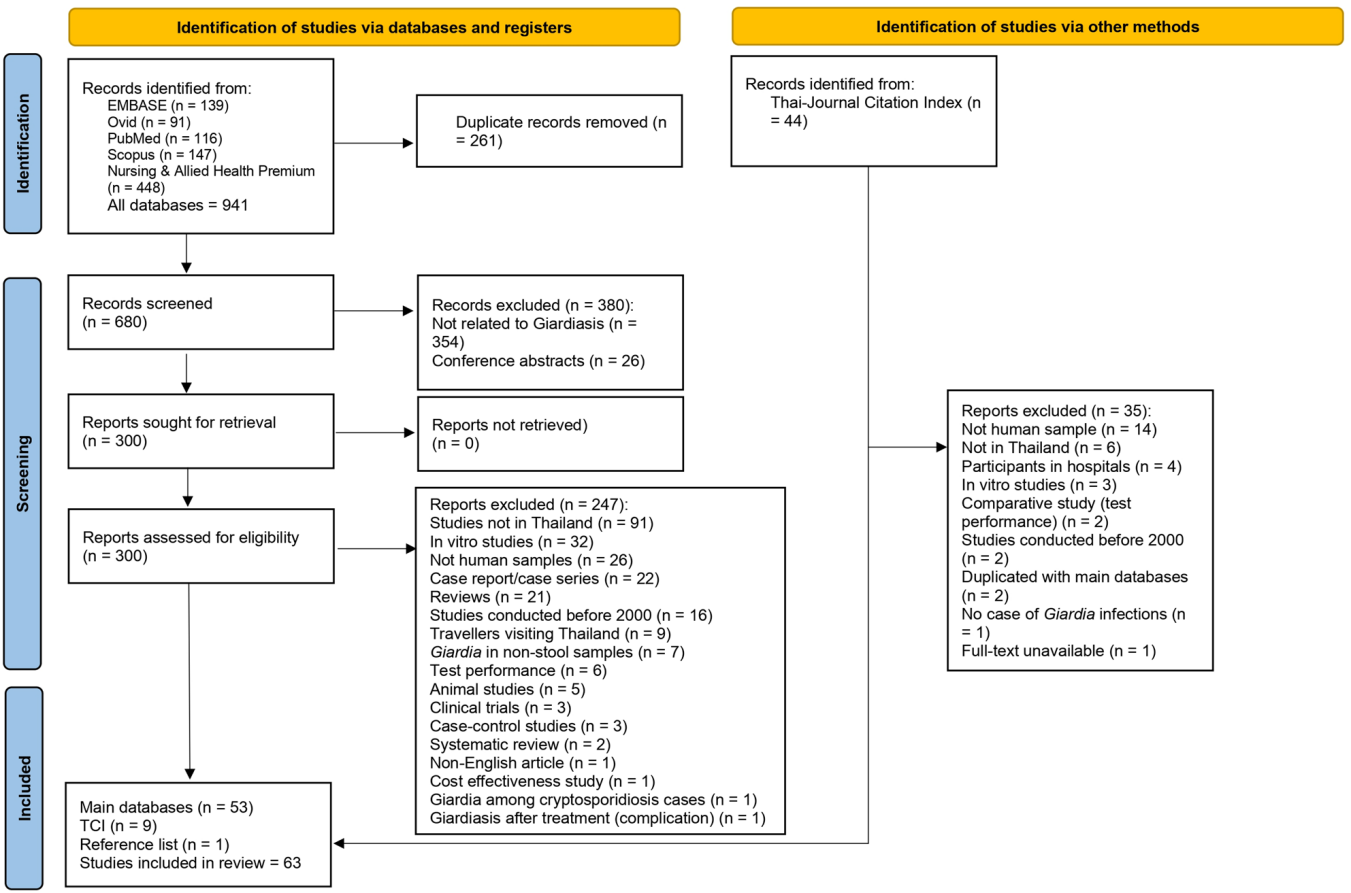
The PRISMA flow diagram. The PRISMA flow diagram outlines the study selection process for the systematic review and meta-analysis of *Giardia duodenalis* infections in Thailand

A total of 941 records were initially identified from EMBASE, Ovid, PubMed, Scopus, Nursing & Allied Health Premium, and the TCI. After removing 261 duplicate records, 680 studies remained for screening. Of these, 380 studies were excluded due to irrelevance or being conference abstracts, leaving 300 full-text reports for eligibility assessment. Further evaluation led to the exclusion of 247 studies for reasons such as being conducted outside Thailand, using non-human samples, being in vitro studies, or focusing on test performance rather than prevalence, leaving 53 studies from the main databases. Finally, 63 studies [8–19, 27–77] were included in the systematic review and meta-analysis: 53 from international databases, nine [9] from TCI, and one [1] from the reference lists of selected studies (Fig. 1).

### Summary characteristics of included studies and risk of bias

Most studies were conducted between 2000 and 2009 (42.86%). Although only 19.05% were published between 2020 and 2024, this five-year period showed a similar annual publication rate to that of 2010–2019. The majority of studies (93.65%) followed a cross-sectional design. Geographically, Central Thailand (47.62%) had the highest number of studies, while other regions, such as Eastern, Northeastern, and Southern Thailand, had significantly fewer studies. Regarding study populations, schoolchildren (28.57%), and villagers (17.46%) were the two groups most frequently studied. Studies primarily targeted children (38.10%), with fewer focusing on adults (14.29%). Direct smear/concentration methods (26.98%) were the most commonly used diagnostic approaches for *G. duodenalis* detection, whereas molecular techniques (4.76%), such as polymerase chain reaction (PCR) and sequencing targeting the glutamate dehydrogenase (*gdh*) or *SSU rRNA* genes, were less frequently employed (Table 1, Table S2). For the 61 cross-sectional studies, 43 (70.5%) were assessed as having a low risk of bias, while 18 (29.5%) had a moderate risk. Among the two cohort studies, one had a low risk of bias and the other a moderate risk (Table S3).

**Table 1:** Summary characteristics of included studies

Characteristics	Number of studies (*N* = 63)	%
2000–2009	27	42.86
2010–2019	24	38.10
2020–2024	12	19.05
**Study design**		
Cross-sectional study	61	96.8
Cohort study	2	3.17
**Regions of Thailand**		
Central Thailand	30	47.62
Northern Thailand	8	12.70
Western Thailand	7	11.11
Eastern Thailand	4	6.35
Northeastern Thailand	4	6.35
Southern Thailand	4	6.35
Central Thailand, Northern Thailand	2	3.17
Central Thailand, Northeastern, Northern, Eastern, Western, Southern Thailand	1	1.59
Central Thailand, Western Thailand	1	1.59
Central Thailand, Eastern Thailand	1	1.59
Not reported	1	1.59
**Types of participants**		
School children	18	28.57
Villagers	11	17.46
Participants with any disease	6	9.52
Hill-tribe children	4	6.35
Orphans	3	4.76
Preschool children	2	3.17
Thai laborers	2	3.17
Migrant workers	2	3.17
Participants who enrolled in health check-up programs	2	3.17
Military personnel	1	1.59
Orphans, childcare workers	1	1.59
School children, adults in the community	1	1.59
Mentally handicapped people	1	1.59
School children, preschool children	1	1.59
Monks or nuns, villagers	1	1.59
Food handlers	1	1.59
Orphans, hill-tribe children	1	1.59
Hill-tribe children, school children	1	1.59
Immigrant children	1	1.59
Refugees	1	1.59
Monks	1	1.59
Participants from the temple community	1	1.59
**Age groups**		
Children	24	38.10
Adults	9	14.29
Children and adults	18	28.57
Not specified	12	19.05
**Detection methods for** ***G. duodenalis***		
Direct smear/concentration methods	17	26.98
Concentration method	16	25.40
Direct smear/concentration methods/molecular method	7	11.11
Direct smear	7	11.11
Direct smear/concentration methods/culture	4	6.35
Molecular method	3	4.76
Direct smear/molecular method	2	3.17
Concentration method/molecular method	1	1.59
Direct smear/culture	1	1.59
Direct smear/immunoassay	1	1.59
Direct smear/concentration methods/culture/other methods	1	1.59
Direct smear/concentration methods/immunoassay	1	1.59
Direct smear/concentration methods/immunofluorescence test	1	1.59
Direct smear/concentration methods/immunofluorescence test/immunoassay	1	1.59

### Prevalence of *Giardia duodenalis* infections in Thailand

**Fig. 2:**
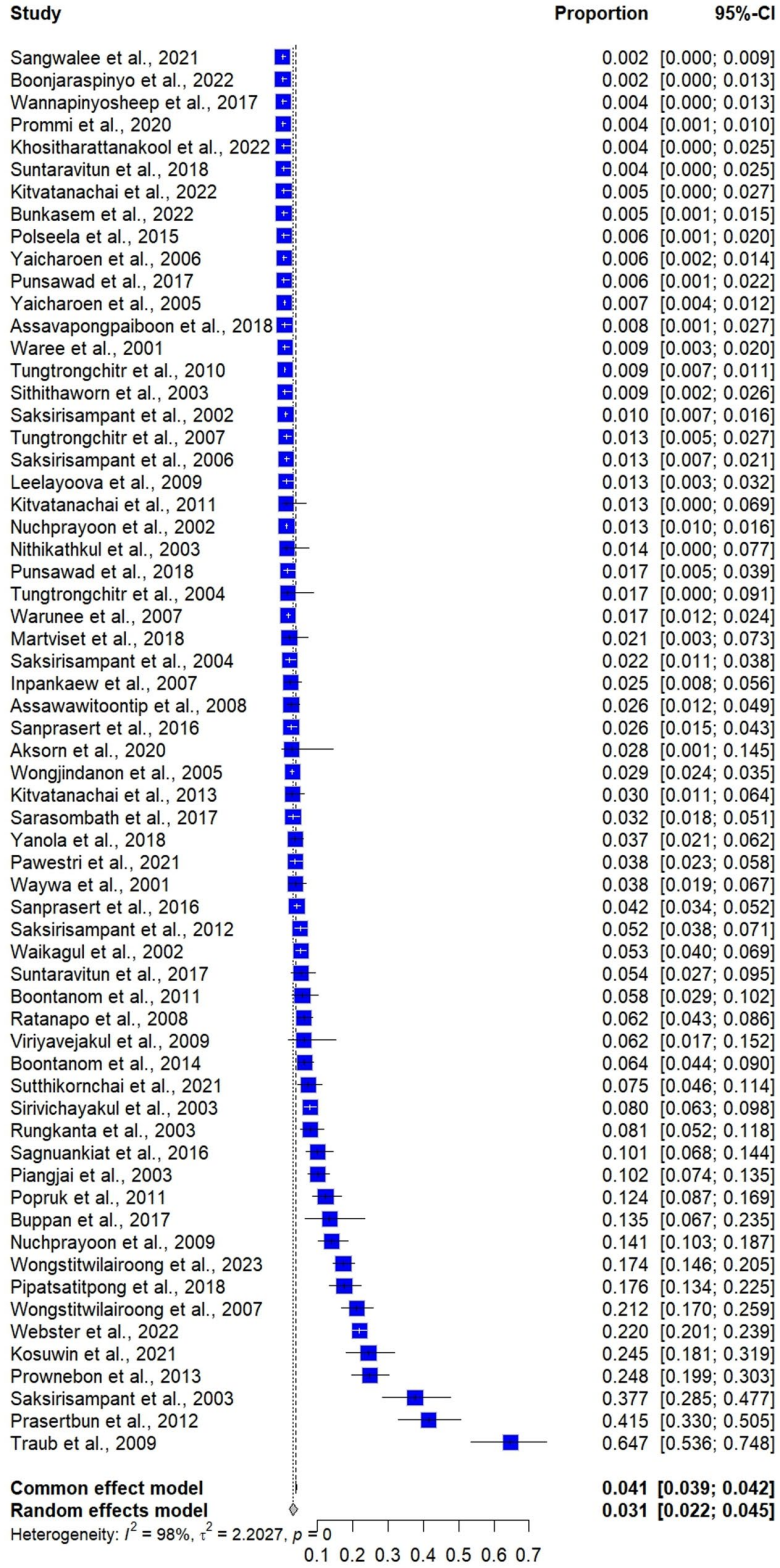
The forest plot illustrates the estimated prevalence of *Giardia duodenalis* infections in Thailand (prevalence estimate = proportion × 100). Each blue square represents an individual study, with its size corresponding to the study’s weight in the meta-analysis. The horizontal lines extending from the squares indicate the 95% confidence interval (CI) for each study’s prevalence estimate, while the vertical dashed line represents the overall effect estimate

**Fig. 3:**
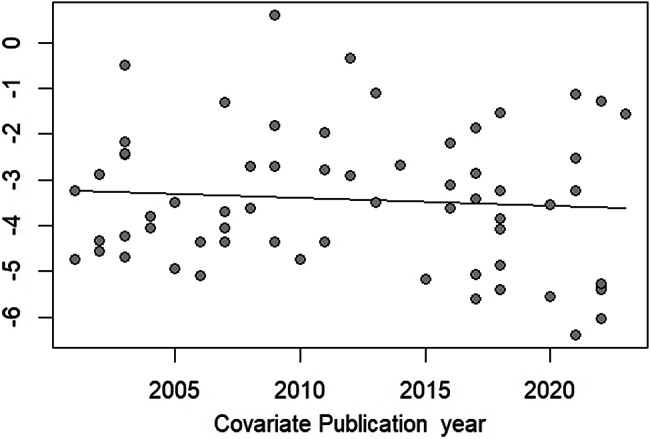
The bubble plot illustrates the distribution of *Giardia duodenalis* infection prevalence estimates in Thailand over time (log-prevalence estimates). Each bubble represents a study, with the X-axis denoting the publication year and the Y-axis representing the log-prevalence estimate. The trend line indicates the overall pattern observed in the data

The meta-analysis using random-effects models showed a prevalence estimate of *G. duodenalis* infections in Thailand at 3.13% (95% CI: 2.16–4.53, *I*^*2*^: 97.7%, number of studies: 63, number of participants: 47,989, and number of *G. duodenalis* infections: 1,945, Fig. 2). The meta-regression analysis indicated that the substantial heterogeneity in prevalence estimates may be attributed to variations in geographical regions of Thailand (*P* = 0.0163), participant types (*P* < 0.001), and detection methods for *G. duodenalis* (*P* = 0.0004) (Table S4). There were slightly lower trends of *G. duodenalis* infections in Thailand from 2000 to 2024, but no significant trends were observed (Fig. 3).

Subgroup analyses revealed no significant differences in prevalence estimates across different publication periods (*P* = 0.599), with prevalence rates ranging from 1.88% (2020–2024) to 3.54% (2010–2019). Prevalence varied significantly by study design (*P* = 0.0004). Cohort studies (prevalence at baseline study) reported the highest prevalence (5.93%), while retrospective descriptive studies had the lowest prevalence (1.46%). Cross-sectional studies, the most commonly used study design, showed a prevalence of 3.19%. *G. duodenalis* prevalence varied significantly across different regions of Thailand (*P* < 0.0001). Western Thailand had the highest prevalence (12.22%), whereas Northeastern Thailand had the lowest prevalence (0.54%). The prevalence of *G. duodenalis* infections differed significantly among participant types (*P* < 0.0001). The highest prevalence based on the subgroup that included at least two studies was observed among orphans (15.14%) and preschool children (11.73%). In contrast, the lowest prevalence was reported in Thai laborers (0.91%) and participants undergoing annual check-ups (0.84%). A significant difference in prevalence was noted between children (5.79%) and adults (0.88%) (*P* = 0.0075), with children showing a considerably higher infection rate.

**Fig. 4:**
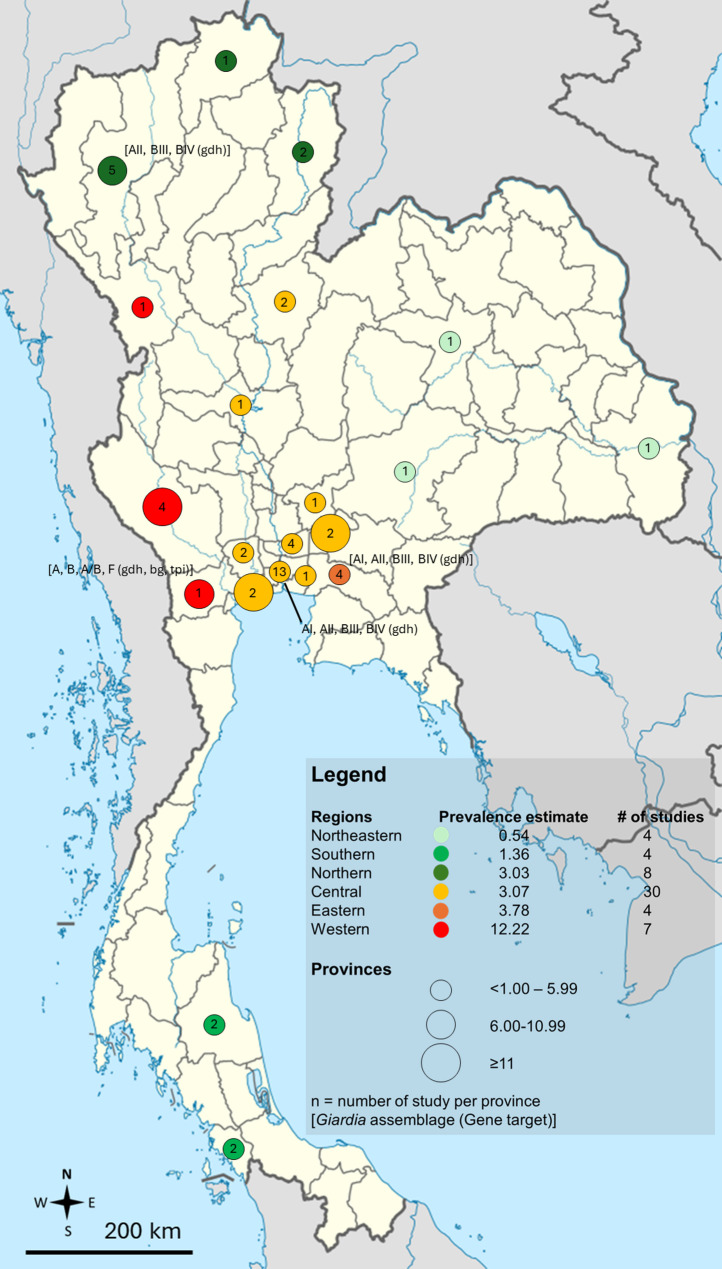
Geographic distribution of the prevalence estimates of *Giardia duodenalis* assemblages in regions and provinces in Thailand. The results are based solely on single-province studies. Data from multi-province studies are excluded from this figure

**Fig. 5:**
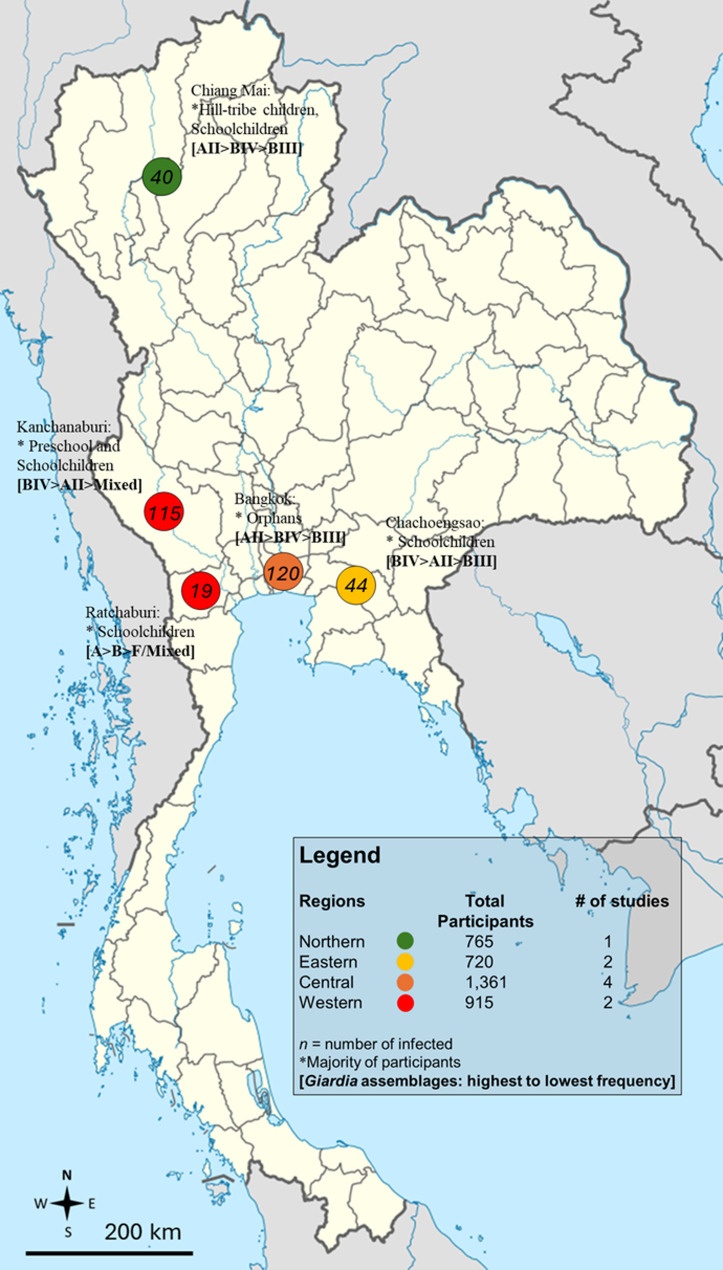
Geographic distribution of the *Giardia duodenalis* subtypes/assemblages reported in Thailand. Data from multi-province studies are excluded from this figure. Genes targeted by molecular methods employed: **gdh** glutamate dehydrogenase; **bg** beta-giardin; **tpi** triosephosphate isomerase

Because the included studies employed heterogeneous diagnostic techniques with varying sensitivities, a stratified meta-analysis was conducted by diagnostic method, rather than combining all estimates into a single overall prevalence. The subgroup analysis revealed significant differences in prevalence estimates of *G. duodenalis* infections depending on the diagnostic method employed (*P* < 0.0001). Studies using only direct smear reported the lowest pooled prevalence (1.40%, 95% CI 0.56–3.46), while those employing concentration methods alone or in combination with direct smear showed slightly higher prevalence (1.99%, 95% CI 1.31–3.00). Culture-based methods produced a pooled estimate of 3.66% (95% CI 0.88–13.92). In contrast, studies incorporating immunoassays with concentration methods yielded markedly higher prevalence (13.44%, 95% CI 5.07–31.07), and molecular techniques, either alone or combined with other methods, showed a higher prevalence of 8.98% (95% CI 4.50–17.14) (Table 2). The prevalence of *G. duodenalis* infections in Thailand stratified by province is presented in Fig. 4 and Table S4. The distribution of *G. duodenalis* assemblages, based on 240 positive samples, is shown in Table 3. Assemblage B was the most common, with a crude proportion of 55.0%, followed by Assemblage A at 32.5%. Mixed infections with Assemblages A and B accounted for 12.08% of cases, and a single case of Assemblage F (0.42%) was also reported (Fig. 5).

**Table 2:** Subgroup analysis of *Giardia duodenalis* infection in Thailand

Parameters	Subgroups	Test for subgroup difference	Prevalence estimate (95% CI)	I^2^ (%)	Number of studies
Overall					
Publication year		0.5990			
	2000–2009		3.42 (2.01–5.74)	97.6	27
	2010–2019		3.54 (2.05–6.06)	97.0	24
	2020–2024		1.88 (0.60–5.77)	96.1	12
Study design		0.0004			
	Cross-sectional study		3.19 (2.15–4.71)	97.8	59
	Cohort study		5.93 (4.22–8.26)	45.2	2
	Retrospective descriptive study		1.46 (0.76–2.79)	82.0	2
Regions of Thailand		< 0.0001			
	Central Thailand		3.07 (1.75–5.35)	97.4	30
	Northern Thailand		3.03 (1.41–6.42)	89.8	8
	Western Thailand		12.22 (5.67–24.4)	95.6	7
	Eastern Thailand		3.78 (1.68–8.25)	57.3	4
	Northeastern Thailand		0.54 (0.23–1.27)	42.3	4
	Southern Thailand		1.36 (0.60–3.04)	63.2	4
	Central Thailand, Northern Thailand		9.02 (1.86–34.16)	99.5	2
	Central Thailand, Northeastern, Northern, Eastern, Western, and Southern Thailand		4.24 (3.43–5.25)	N/A	1
	Central Thailand, Western Thailand		0.88 (0.68–1.12)	N/A	1
	Central Thailand, Eastern Thailand		2.61 (1.58–4.29)	N/A	1
	Not reported		7.96 (6.43–9.81)	N/A	1
Types of participants		< 0.0001			
	School children		3.17 (1.87–5.32)	95.5	18
	Villagers		1.04 (0.42–2.53)	95.0	11
	Participants with any disease		2.05 (1.18–3.54)	78.9	6
	Hill-tribe children		5.20 (2.83–9.39)	89.8	4
	Orphans		15.14 (6.01–33.22)	96.9	3
	Preschool children		11.73 (4.51–27.22)	94.7	2
	Thai laborers		0.91 (0.61–1.34)	49.4	2
	Migrant workers		1.62 (0.05–33.62)	95.1	2
	Participants who enrolled in health check-up programs		0.84 (0.67–1.05)	0.0	2
	Military personnel		1.26 (0.47–3.31)	N/A	1
	Orphans, childcare workers		17.65 (13.67–22.48)	N/A	1
	School children, adults in the community		0.51 (0.17–1.58)	N/A	1
	Mentally handicapped people		7.96 (6.43–9.81)	N/A	1
	School children, preschool children		17.40 (14.69–20.48)	N/A	1
	Monks or nuns, villagers		2.45 (1.02–5.75)	N/A	1
	Food handlers		1.27 (0.18–8.44)	N/A	1
	Orphans, hill-tribe children		24.82 (20.13–30.20)	N/A	1
	Hill-tribe children, school children		5.23 (3.86–7.05)	N/A	1
	Immigrant children		10.11 (7.03–14.34)	N/A	1
	Refugees		21.96 (20.13–23.91)	N/A	1
	Monks		2.78 (0.39–17.26)	N/A	1
	Participants from the temple community		64.71 (54.02–74.10)	N/A	1
Age groups		0.0075			
	Children		5.79 (3.58–9.26)	96.6	24
	Adults		0.88 (0.31–2.51)	94.9	9
	Children and adults		02.43 (1.09–5.33)	98.5	18
	Not specified		3.00 (1.61–5.53)	97.2	12
Detection methods for *G. duodenalis*		< 0.0001			
	Concentration alone or with direct smear		1.99 (1.31–3.00)	94.1	33
	Concentration methods with immunoassays (with or without direct smear)		13.44 (5.07–31.07)	95.0	3
	Culture alone or with non-molecular methods		3.66 (0.88–13.92)	97.2	6
	Molecular method alone or with other methods		8.98 (4.50–17.14)	98.6	14
	Direct smear only		1.40 (0.56–3.46)	94.1	7

N/A, not assessed

**Table 3:** Distribution of *Giardia duodenalis* assemblages in Thailand (total positive samples = 240)

Assemblage	No. of studies	Observed (*n*)	Crude proportion (%)
A	10	78	32.5
B	10	132	55.0
A/B (mixed)	3	29	12.08
F	1	1	0.42

Notes: –, unable to calculate pooled proportion estimate due to limited data

N/A, not assessed

### Sensitivity analysis and publication bias

**Fig. 6:**
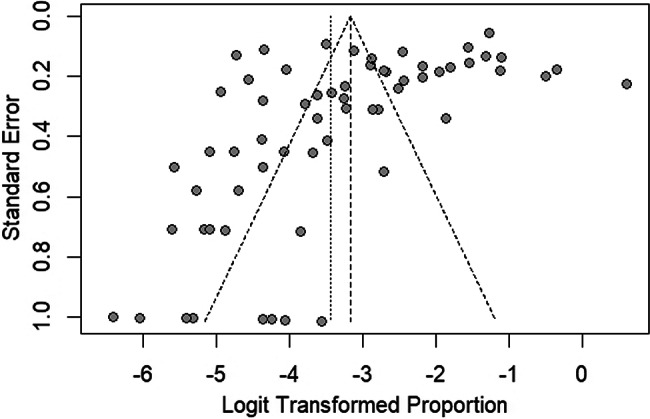
The funnel plot. The funnel plot evaluates publication bias in the meta-analysis by plotting the standard error against the logit-transformed proportion of *Giardia duodenalis* infections in Thailand. Each gray dot represents a prevalence estimate from an individual study. An asymmetrical distribution of points around the central line may indicate potential bias in the included studies

The fixed-effect model estimated the prevalence of *G. duodenalis* infections in Thailand at 4.05% (95% CI: 3.88–4.53, Fig. 2). The funnel plot assessing publication bias in the meta-analysis reveals an uneven distribution of points around the central line, indicating potential bias in the included studies (Fig. 6). Egger’s regression test further confirms significant asymmetry (*P* = 0.0011), suggesting the presence of publication bias in the prevalence estimate analysis.

## Discussion

The current meta-analysis provides a comprehensive overview of the prevalence and distribution of *G. duodenalis* infections in Thailand over the past two decades. The results indicated a slight but non-significant declining trend from 2000 to 2024, suggesting that *G. duodenalis* remains an endemic issue in Thailand. The meta-analysis revealed an overall prevalence of 3.13%, with significant heterogeneity among the included studies. This variability is likely influenced by differences in geographical regions, study populations, and diagnostic methods. The subgroup analysis highlighted distinct prevalence patterns based on participant characteristics. Based on the subgroups with similar participants in studies, orphans (15.14%) and preschool children (11.73%) appear to have a higher prevalence than villagers (1.04%) and school children (3.17%). This disparity aligns with previous studies indicating that children are more susceptible to *G. duodenalis* infections due to behavioral factors, such as exposure to contaminated environments, hand-to-mouth activities, poor toilet training, and poor food-handling hygiene [2, 78]. Other plausible reasons for high prevalence among orphans in addition to poor living conditions include limited supervision of personal hygiene, insufficient access to clean water, and inadequate sanitation infrastructure in institutional settings [9, 10]. Similarly, high prevalence rates observed among hill-tribe children, immigrant children, and refugees likely reflect the combined influence of crowding, poor sanitation, and restricted access to safe water and healthcare services. The prevalence of *G. duodenalis* infections among school children in this study appears relatively low at 3.17%, compared to reports from other developing countries, where rates often exceed 10% in high-risk populations. Specifically, among children, the prevalence of *G. duodenalis* infections in Thailand is considerably lower compared to estimates from other regions synthesized by meta-analysis approaches, such as 15.1% among Asian children [7] and 18.3% among African children [6]. These results suggest that schools in Thailand are promoting less environmental contamination and better hygiene practices, which may be attributed to the availability of facilities that prevent *G. duodenalis* infection, such as adequate toilets, soap, and access to safe drinking water. These improvements may be partly attributed to the implementation of Thailand’s Water, Sanitation, and Hygiene (WASH) program, which aims to enhance access to safe water, adequate sanitation, and hygiene education, especially in schools and vulnerable communities.ualit

Among patients with underlying diseases, the prevalence of *G. duodenalis* infections in this study was 2.05%. This group included individuals with nephrotic syndrome undergoing renal biopsy, HIV patients receiving antiretroviral therapy, patients infected with HIV, acquired immunodeficiency syndrome (AIDS) patients with diarrhea, and those with irritable bowel syndrome. A previous meta-analysis estimated the global prevalence of *G. duodenalis* infection in cancer patients at 6.9% [79]. Similarly, a meta-analysis of *G. duodenalis* infection in Africa reported a pooled prevalence of 8.8%, with a lower prevalence in HIV patients (5.0%) compared to those with diarrheal symptoms (12.3%) [80]. These findings suggest that *G. duodenalis* infection rates are more influenced by environmental contamination and hygiene practices rather than the presence of other diseases or co-infections. Nevertheless, HIV infection may increase the risk of specific *G. duodenalis* assemblages, such as Assemblage B, but not Assemblage A [81]. Additionally, a recent meta-analysis demonstrated that HIV/AIDS patients with diarrhea were nearly four times more likely to have *G. duodenalis* infections than those without diarrhea [82]. These data suggest that individuals with other diseases accompanied by diarrhea symptoms may have an increased risk of *G. duodenalis* infection. Although *G. duodenalis* is classically recognized as an enteric pathogen capable of causing acute or chronic diarrhea, the large multicounty study showed that many infections are asymptomatic as *G. duodenalis* was frequently detected in both diarrheal and non-diarrheal stool samples from young children across diverse low- and middle-income countries [83]. Emerging evidence suggests that underlying diseases and co-infections may influence *G. duodenalis* colonization by altering gut microbiota composition [84–86]. *G. duodenalis* infection itself can induce changes in the commensal microbiota, known as dysbiosis, which may contribute to variations in the clinical manifestations of giardiasis [84]. Additionally, *G. duodenalis* infection can facilitate bacterial translocation by disrupting the intestinal epithelium and compromising tight junction integrity [85, 86]. Experimental studies in mice have shown that infection with Assemblage AII induces more pronounced leukocytosis than Assemblage BIV [87], suggesting that Assemblage AII may exhibit greater pathogenic potential. This could partly explain the assemblage-specific prevalence patterns observed in certain immunocompromised populations.

The subgroup analysis also revealed regional differences in the prevalence of *G. duodenalis* infection in Thailand, with Western Thailand reporting the highest prevalence (12.22%) and Northeastern Thailand the lowest (0.54%). These findings suggest that more urbanized regions, such as Central Thailand, where most of the included studies in this systematic review were conducted, may have a relatively lower prevalence (3.07%) due to better sanitation and access to healthcare. In contrast, the higher prevalence in Western Thailand likely reflects the rural and border areas (along the Thai-Myanmar border), where reliance on surface water, limited sanitation, and frequent migration contribute to transmission. However, it is important to note that almost 70% of participants in the Western Thailand studies were children, whereas none of the Northeastern Thailand studies included children. Since children are known to have a significantly higher risk of *G. duodenalis* infection, the apparent regional differences may largely reflect differences in the age distribution of study populations rather than true geographical variation. In addition, other contextual factors may still contribute. Western Thailand, characterized by dense forests, rivers, and shared borders with Myanmar, experiences frequent migration and cross-border movement, which may increase exposure to contaminated water and food, poor personal hygiene, inadequate sanitation, and close contact with animals, factors that could elevate the risk of *G. duodenalis* transmission. In contrast, Northeastern Thailand has a drier climate and relies more on underground water sources, less prone to surface contamination. Nonetheless, the influence of population age structure as a confounder should be considered when interpreting these regional differences. The higher prevalence in Western Thailand may also reflect the greater number of studies using sensitive diagnostic methods (e.g., molecular techniques), which are more effective at detecting *G. duodenalis*. Meanwhile, Northeastern Thailand had fewer studies and may have relied on less sensitive diagnostic methods, potentially leading to an underestimation of prevalence.

The subgroup analysis showed that the diagnostic approach significantly influenced prevalence estimates, with the lowest estimates observed when traditional microscopic methods (direct smear or concentration) were used, and substantially higher estimates obtained with immunoassays or molecular techniques. The subgroup analysis showed that the diagnostic approach significantly influenced prevalence estimates, with the lowest estimates observed when traditional microscopic methods (direct smear or concentration) were used, and substantially higher estimates obtained with immunoassays or molecular techniques. This difference likely reflects the greater sensitivity of molecular tools, which can detect *G. duodenalis* even at low parasite loads [88–90], whereas conventional microscopy may underestimate the true burden in Thailand. However, despite their superior performance, molecular techniques remain underutilized in Thailand, where conventional microscopy-based methods are still more commonly applied. This restricts opportunities to generate valuable data on *Giardia* species/assemblage distribution, which is critical for understanding host specificity and transmission sources. Notably, only a few studies employed both molecular and conventional methods within the same population, limiting direct comparison of their performance. While the current findings highlight the diagnostic advantages of molecular methods, their wider adoption in surveillance and research is necessary to avoid underestimating the true burden of *G. duodenalis* infections.

Regarding the distribution of *G. duodenalis* assemblages in Thailand, analysis of 240 positive samples showed that Assemblage B was the most common (55.0%), followed by Assemblage A (32.5%). Mixed infections with Assemblages A and B accounted for 12.08% of cases, and a single case of Assemblage F (0.42%) was also identified (Table 3). This distribution is in line with global trends, where Assemblage B frequently predominates in human infections [91]. Assemblages A and B primarily infect humans and a range of other mammals, whereas assemblages C–H are mainly detected in non-human animal hosts [92]. Although the reasons for the predominance of Assemblage B remain unclear, it may be partly related to higher cyst shedding rates compared to Assemblage A, which could facilitate transmission [93]. Assemblage A is often associated with symptomatic infections and acute diarrheal illness, whereas Assemblage B has been linked to more prolonged or asymptomatic infections in some populations [92, 93]. However, the evidence is not entirely consistent, as several studies have found the opposite trend or no clear association between assemblage type and symptom severity [91, 92]. The detection of Assemblage F in a human sample, though rare, is noteworthy because this genotype is typically associated with cats and dogs [94]. Moreover, while Assemblage E was not identified in any of the included studies from Thailand, it has been sporadically reported in humans in Brazil [95], Egypt [96], Australia [97], and Vietnam [98], suggesting a potential zoonotic route of *G. duodenalis* transmission. Such host-adapted assemblages occasionally infect humans [99], reflecting the complex interactions among host, parasite, and environment. These findings suggest that both zoonotic and anthroponotic transmission pathways likely contribute to the spread of *G. duodenalis* in Thailand. Consequently, molecular epidemiological surveillance that incorporates assemblage typing is essential for distinguishing human- and animal-derived infections, identifying environmental reservoirs, and designing targeted control strategies. Strengthening molecular data collection will enhance the understanding of local transmission dynamics and inform public health interventions aimed at reducing the risk of giardiasis in both humans and animals. From a public health perspective, these findings underscore the need for local programs focused on case detection and monitoring treatment outcomes, given that chronic *G. duodenalis* infection can lead to both intestinal and extra-intestinal complications, including ocular pathologies, arthritis, allergies, impaired growth, cognitive deficits, and post-infectious irritable bowel syndrome [87].

The findings of this study have important public health implications for Thailand. The consistently higher prevalence observed among orphans, preschool children, and hill-tribe or migrant communities indicates that *G. duodenalis* transmission is linked to social vulnerability and living conditions rather than being uniformly distributed across the population. Likewise, the markedly higher prevalence in Western Thailand compared to Northeastern Thailand suggests that regional environmental and socio-demographic factors, such as reliance on surface water, frequent migration, and cross-border movement, may play key roles in transmission. These insights highlight the need for context-specific interventions rather than generic approaches. For example, in high-prevalence areas, *Giardia* surveillance could be integrated into child health programs and school-based hygiene initiatives. In contrast, in migrant and orphanage settings, enhanced screening and improved sanitation infrastructure may be more effective.

Although this systematic review provides the most comprehensive synthesis of *G. duodenalis* data in Thailand to date, the substantial statistical heterogeneity (*I²* = 97.7%) among studies warrants emphasis. The pooled prevalence estimate should be interpreted with caution, as it represents an average across highly diverse methodologies and epidemiological contexts rather than a precise national figure. Stratified analyses by region, participant type, and diagnostic method, therefore, offer a more meaningful interpretation of *G. duodenalis* epidemiology in Thailand. Similarly, the molecular data included in this systematic review were limited, with assemblage distribution derived from only 240 positive samples analyzed by molecular techniques, representing a small fraction of all *G. duodenalis*-positive cases. Consequently, conclusions regarding assemblage predominance and zoonotic transmission potential should also be viewed cautiously. Expanded molecular surveillance and standardized genotyping protocols are crucial for generating more representative assemblage data and elucidating transmission pathways across diverse ecological and demographic contexts.

## Conclusion

This systematic review and meta-analysis revealed that the prevalence of *G. duodenalis* infection in Thailand varies markedly by diagnostic method, with molecular and immunoassay-based studies detecting substantially higher rates than traditional microscopy. Although the pooled prevalence provides a national overview, the high heterogeneity among studies limits its precision and should be interpreted with caution. The evidence indicates that *G. duodenalis* remains endemic, particularly among socially vulnerable groups such as orphans, preschool children, and migrant or hill-tribe communities. Regional variation, with the highest prevalence in Western Thailand, suggests that environmental factors, water sources, and population mobility contribute to local transmission. Molecular data revealed a predominance of Assemblage B, followed by Assemblage A, but the limited number of molecularly characterized samples restricts the representativeness of these findings. Strengthening surveillance with sensitive molecular tools and adopting a One Health approach that integrates human, animal, and environmental monitoring will be crucial in guiding targeted interventions and mitigating giardiasis transmission in Thailand.

## Supplementary Information

Below is the link to the electronic supplementary material.

Supplementary Material 1

Supplementary Material 2

Supplementary Material 3

Supplementary Material 4

Supplementary Material 5

## Data Availability

All data related to the present study are available in this manuscript, as well as in the Table S1, Table S2, Table S3, and Table S4 files.

## Abbreviations

AIDS: Acquired immunodeficiency syndrome
CIs: Confidence intervals
HIV: Human immunodeficiency virus
JBI: Joanna Briggs Institute
PRISMA: Preferred Reporting Items for Systematic Reviews and Meta-Analyses
SSU rRNA: Small subunit ribosomal ribonucleic acid
TCI: Thai-Journal Citation Index
