# Supplementary material for: Prevalence and genotyping of Giardia duodenalis infections in humans in Thailand: a systematic review and meta-analysis
Source: BMC Infect Dis. 2025 Dec 20;26:131. doi: 10.1186/s12879-025-12372-6 (PMC12831254; doi:10.1186/s12879-025-12372-6)
Supplement: Supplementary file 1 — Supplementary Material 1 [file 12879_2025_12372_MOESM1_ESM.docx]

**Table S1. Search terms**

**General keywords**

(Giardia OR Giardias OR Giardiasis OR “Giardia lamblia” OR Lamblia OR Lamblias OR “Giardia intestinalis” OR duodenalis OR “Giardia duodenalis”) AND (Thailand OR Siam)

PubMed 18 December 2024

| No. | Key concept | Search terms | Results |
| --- | --- | --- | --- |
| 1. | Giardia | Giardia[Text Word] OR Giardias[Text Word] OR Giardiasis[Text Word] OR “Giardia lamblia”[Text Word] OR Lamblia[Text Word] OR Lamblias[Text Word] OR “Giardia intestinalis”[Text Word] OR duodenalis[Text Word] OR “Giardia duodenalis”[Text Word] OR Giardia[MeSH Terms] OR “Giardia lamblia”[MeSH Terms] OR “Giardia intestinalis”[MeSH Terms] OR “Giardia duodenalis”[MeSH Terms] | 12,947 |
| 2. | Thailand | Thailand[Text Word] OR Thailand[MeSH Terms] OR Siam[Text Word] OR Siam[MeSH Terms] | 48,013 |
| 3. | #1 AND #2 | (Giardia[Text Word] OR Giardias[Text Word] OR Giardiasis[Text Word] OR “Giardia lamblia”[Text Word] OR Lamblia[Text Word] OR Lamblias[Text Word] OR “Giardia intestinalis”[Text Word] OR duodenalis[Text Word] OR “Giardia duodenalis”[Text Word] OR Giardia[MeSH Terms] OR “Giardia lamblia”[MeSH Terms] OR “Giardia intestinalis”[MeSH Terms] OR “Giardia duodenalis”[MeSH Terms]) AND (Thailand[Text Word] OR Thailand[MeSH Terms] OR Siam[Text Word] OR Siam[MeSH Terms]) | 116 |

Embase 18 December 2024

| No. | Key concept | Search terms | Results |
| --- | --- | --- | --- |
| 1. | Giardia | Giardia:ti,ab,kw,de OR Giardias:ti,ab,kw,de OR Giardiasis:ti,ab,kw,de OR “Giardia lamblia”:ti,ab,kw,de OR Lamblia:ti,ab,kw,de OR Lamblias:ti,ab,kw,de OR “Giardia intestinalis”:ti,ab,kw,de OR duodenalis:ti,ab,kw,de OR “Giardia duodenalis”:ti,ab,kw,de OR Giardia/exp OR “Giardia lamblia”/exp OR “Giardia intestinalis”/exp OR “Giardia duodenalis”/exp | 18,438 |
| 2. | Thailand | Thailand:ti,ab,kw,de OR Thailand/exp OR Siam:ti,ab,kw,de OR Siam/exp | 55,994 |
| 3. | #1 AND #2 | (Giardia:ti,ab,kw,de OR Giardias:ti,ab,kw,de OR Giardiasis:ti,ab,kw,de OR “Giardia lamblia”:ti,ab,kw,de OR Lamblia:ti,ab,kw,de OR Lamblias:ti,ab,kw,de OR “Giardia intestinalis”:ti,ab,kw,de] OR duodenalis:ti,ab,kw,de OR “Giardia duodenalis”:ti,ab,kw,de OR Giardia/exp OR “Giardia lamblia”/exp OR “Giardia intestinalis”/exp OR “Giardia duodenalis”/exp) AND (Thailand:ti,ab,kw,de OR Thailand/exp OR Siam:ti,ab,kw,de OR Siam/exp) | 139 |

Scopus 18 December 2024

| No. | Key concept | Search terms | Results |
| --- | --- | --- | --- |
| 1. | Giardia | TITLE-ABS-KEY ( giardia OR giardias OR giardiasis OR "Giardia lamblia" OR lamblia OR lamblias OR "Giardia intestinalis" OR duodenalis OR "Giardia duodenalis" ) | 19,438 |
| 2. | Thailand | TITLE-ABS-KEY ( thailand OR siam ) | 129,585 |
| 3. | 1 AND 2 | ( TITLE-ABS-KEY ( giardia OR giardias OR giardiasis OR "Giardia lamblia" OR lamblia OR lamblias OR "Giardia intestinalis" OR duodenalis OR "Giardia duodenalis" ) ) AND ( TITLE-ABS-KEY ( thailand OR siam ) ) | 147 |

Ovid 18 December 2024

| No. | Key concept | Search terms | Results |
| --- | --- | --- | --- |
| 1. | Giardia AND Thailand | (Giardia OR Giardias OR Giardiasis OR "Giardia lamblia" OR Lamblia OR Lamblias OR "Giardia intestinalis" OR duodenalis OR "Giardia duodenalis") AND (Thailand OR Siam) {Including Limited Related Terms} | 91 |

ProQuest 18 December 2024

| No. | Key concept | Search terms | Results |
| --- | --- | --- | --- |
| 1. | Giardia AND Thailand | (Blastocystis OR Blastocysti OR blastocystina OR "Blastocystis hominis") AND (Thailand OR Siam) | 449 |

Thai-Journal Citation Index 18 December 2024

| No. | Key concept | Search terms | Results |
| --- | --- | --- | --- |
| 1. | Giardia AND Thailand | Giardia | 44 |

TCI selection

- Not in Thailand (n = 6)
- Not human sample (n = 14)
- Comparative study (test performance) (n = 2)
- No Giardia case (n = 1)
- In vitro study (n = 3)
- Full-text unavailable (n = 1)
- Duplicated with main databases (n = 2)
- Conducted before 2000 (n = 2)
- Participants in hospitals (n = 4)

Final included (n = 9)
