# Supplementary material for: Prevalence and genotyping of Giardia duodenalis infections in humans in Thailand: a systematic review and meta-analysis
Source: BMC Infect Dis. 2025 Dec 20;26:131. doi: 10.1186/s12879-025-12372-6 (PMC12831254; doi:10.1186/s12879-025-12372-6)
Supplement: Supplementary file 4 — Supplementary Material 4 [file 12879_2025_12372_MOESM4_ESM.docx]

**Table S4.** Meta-regression and subgroup analysis

**1. Meta-regression analysis of the prevalence of *Giardia* infections in Thailand**

| **Covariates** | **tau^2^** | **Test for residual heterogeneity, *P* value** | **Residual heterogeneity *I^2^* (%)** | **Test of moderators, *P* value** | **Number of studies** |
| --- | --- | --- | --- | --- | --- |
| Publication years | 2.2013 | < 0.001 | 98.09 | 0.5420 | 63 |
| Study design | 2.1791 | < 0.001 | 98.21 | 0.7978 | 63 |
| Regions of Thailand | 1.5862 | < 0.001 | 96.89 | 0.0163 | 63 |
| Age groups | 2.1791 | < 0.001 | 98.21 | 0.7978 | 63 |
| Male percentage | 2.0303 | < 0.001 | 97.99 | 0.2078 | 47 |
| Types of participants | 0.8827 | < 0.001 | 93.22 | < 0.001 | 63 |
| Method for *Giardia* detection | 1.5806 | < 0.001 | 97.29 | 0.0002 | 63 |

**2. Subgroup analysis of the prevalence of *Giardia* infections in Thailand (by province)**

| **Regions of Thailand** | **Province** | **Prevalence estimate (95% CI)** | ***I^2^* (%)** | **Number of studies** |
| --- | --- | --- | --- | --- |
| Overall |  | 3.10 (2.16–4.45) | 97.8 | 63 |
| Eastern Thailand |  | 3.78 (1.68–8.25) | 57.3 | 4 |
|  | Chachoengsao | 3.78 (1.68–8.25) |  | 4 |
| Western Thailand |  | 12.22 (5.67–24.40) | 95.6 | 7 |
|  | Kanchanaburi | 15.39 (4.96–38.81) |  | 4 |
|  | Tak | 3.78 (2.42–5.85) |  | 1 |
|  | Ratchaburi | 7.48 (4.82–11.43) |  | 1 |
| Central Thailand |  | 3.07 (1.75–5.35) | 98.1 | 30 |
|  | Bangkok | 3.86 (01.68–8.64) |  | 13 |
|  | Pathum Thani | 4.56 (0.90–20.00) |  | 4 |
|  | Nakhon Nayok | 19.87 (12.99–29.17) |  | 2 |
|  | Samut Sakhon | 12.16 (9.64–15.22) |  | 2 |
|  | Phitsanulok | 0.75 (0.36–1.56) |  | 2 |
|  | Nakhon Pathom | 1.15 (0.56–2.35) |  | 2 |
|  | Nakhon Sawan | 0.51 (0.17–1.58) |  | 1 |
|  | Samut Prakan | 0.37 (0.09–1.47) |  | 1 |
|  | Saraburi | 0.76 (0.19–2.99) |  | 1 |
| Northern Thailand |  | 3.03 (1.41–6.42) | 89.8 | 8 |
|  | Chiang Rai | 0.45 (0.06–3.10) |  | 1 |
|  | Chiang Mai | 5.23 (3.25–8.33) |  | 5 |
|  | Nan | 1.48 (0.22–9.37) |  | 2 |
| Northeastern Thailand |  | 0.54 (0.23–1.27) | 42.3 | 4 |
|  | Khon Kaen | 0.24 (0.03–1.67) |  | 1 |
|  | Ubon Ratchathani | 1.25 (0.56–2.76) |  | 1 |
|  | Nakhon Ratchasima | 0.17 (0.02–1.17) |  | 1 |
| Southern Thailand |  | 1.36 (0.60–3.04) | 63.2 | 4 |
|  | Satun | 1.68 (0.46–5.95) |  | 2 |
|  | Nakhon Si Thammarat | 1.12 (0.54–2.34) |  | 2 |

*The results in the table are based solely on single-province studies. Data from multi-province studies are excluded from this table.
