# Supplementary material for: Prevalence and genotyping of Giardia duodenalis infections in humans in Thailand: a systematic review and meta-analysis
Source: BMC Infect Dis. 2025 Dec 20;26:131. doi: 10.1186/s12879-025-12372-6 (PMC12831254; doi:10.1186/s12879-025-12372-6)
Supplement: Supplementary file 5 — Supplementary Material 5 [file 12879_2025_12372_MOESM5_ESM.docx]

**Table S5.** Egger’s test for the proportion estimates of *Blastocystis* subtypes in Thailand

| **Subtypes** | ***P* value** | **Bias estimate** | **t** | **df** |
| --- | --- | --- | --- | --- |
| Assemblage A | 0.1194 | -2.8781 | -1.74 | 8 |
| Assemblage B | 0.4175 | -0.9292 | -0.85 | 8 |
